# The role of emotion regulation in normative influence under uncertainty

**DOI:** 10.1186/s40359-025-03033-z

**Published:** 2025-07-04

**Authors:** Magnus Bergquist, Malin Ekelund

**Affiliations:** 1 Department of Psychology, University of Gothenburg, Haraldsgatan 1, Göteborg, 41314 Sweden; 2 Åbo Akademi University, Turku, Finland

**Keywords:** Social norms, Social influence, Decision-making, Uncertainty, Emotion regulation

## Abstract

Social norms are pivotal in guiding decision-making. Motives for following social norms are often described as gaining or avoiding (dis)approval and as an adaptive heuristic. We propose that social norms also serve an emotion-regulating function. Across three preregistered experiments (n_total_ = 2518), we operationalized uncertainty as complexity, risk, and ambiguity. Results first confirmed that descriptive social norms are more influential in uncertain situations than in less uncertain ones. When testing the psychological processes, uncertain situations did not make social norms more salient. For emotion regulation, however, we found a fairly consistent pattern across all three experiments; being exposed to a descriptive social norm mitigated participants' negative emotions. These results advance past research by suggesting that descriptive norms serve an emotion-regulating function.

**Supplementary Information:**

The online version contains supplementary material available at 10.1186/s40359-025-03033-z.

## Introduction

Decision-making often sways in response to the choice of our peers. Descriptive social norms, reflecting how others behave, often play a significant role in shaping both trivial judgments [2] and complex decisions, such as behavioral responses in social dilemmas [10]. Drawing on people's motivation to conform to social norms, research has applied norms-based information to change a wide range of behaviors. These efforts have resulted in healthier lunch choices [54], reduced alcohol consumption [46], lowered household electricity use [51], increased voter turnout [29], and safer sex practices [17]. Meta-analyses demonstrate that referencing social norms generally constitutes an effective strategy to promote healthy and sustainable behaviors [8], 60, 13].

However, it is important to note that this is not always the case. Findings from applied psychology exhibit dispersive effects of social norms, ranging from substantial [5, 36] to nearly negligible e.g., [40, 44], or even negative e.g., [11, 45]. Hence, it is crucial to examine boundary conditions that determine how and when social norms affect decision-making. In this article, we focus on one largely overlooked consequence of social norms, that displaying a descriptive social norm can regulate negative emotions. We propose that social norms might be motivated, not only by avoiding/gaining (dis)approval or as an effective heuristic (i.e., following others'behaviors), but are also a means to regulate negative emotions. Essentially, when being in a negative state of uncertainty, turning to others will provide emotional comfort.

In general, social normative influence operates via two paths: normative versus informational social influence [24], corresponding to injunctive and descriptive social norms [19]. This conceptual separation suggests that people conform to social norms to avoid or gain others (dis)approval, or because other people’s behaviors serve as cues for the “correct” or adaptive behavior in that specific situation [18].

Recent research has broadened those motivational antecedents by emphasizing the role of emotions in conforming to social norms [43]. Although personal norms are both activated and motivated by negative emotions such as guilt and obligations [49, 56] less attention has been given to the role of negative emotions in social normative decision-making. In terms of boundary conditions for social normative influence, however, perceived uncertainty has been described as a central condition under which people are more likely to follow social norms [21]. While uncertainty has shown to be associated with perceived negative emotions [43], to the best of our knowledge, little is known about how turning to social norms might regulate negative emotions induced by uncertainty. Across three experiments, we test and explore if descriptive norms are more likely to shape decision-making under uncertainty, and if descriptive social norms have an emotion-regulating function.

### Decision-making under uncertainty

The umbrella concept of uncertainty, which can be described as the conscious awareness of ignorance, encompasses complexity, risk, and ambiguity [1]. Complexity, as a subdimension, may contribute to perceived uncertainty, as complex situations tend to be more difficult to understand, less predictable, and reduce confidence in decision-making [3]. Uncertainty can also be divided into the psychological constructs of risk, where likelihoods are known, and ambiguity, where likelihoods are unknown [25, 28]. Hence, by uncertainty, we refer to situations where people are made aware of complexity, risk, and/or ambiguity of a decision-making task.

The social comparison theory proposes that people are more likely to seek social comparisons in uncertain situations [26]. Research suggests that social norms are more influential under uncertainty [21]. Meta-analytic evidence of replications of Asch’s line judgment show that more complex stimulus materials amplified social influence [12]. In this case, more subjects conformed to peers’ faulty assessments when perceiving the line judgment task as more uncertain. Another line of evidence emerges from a meta-analysis of the bystander effect, reporting that individuals are more prone to be influenced by others' inaction in uncertain contexts, whereas unambiguously dangerous situations are more likely to prompt decisive actions [27]. Although meta-analytic evidence is often viewed as the gold standard of empirical evidence, subgroup effects should be interpreted with caution, as meta-analysis is an observational method. In these cases, results do not imply causality and might be driven by confounding variables. Evidence from studies using cross-sectional designs reports that the relationship between descriptive norms and behavioral intentions is indeed moderated by uncertainty [39], 47]. Descriptive norms were more closely related to intentions for more uncertain risks (i.e., non-established risk behaviors, such as avoiding BPA plastics and using hands-free headset) than for more certain risks, such as smoking and applying sunscreen [39].

The psychological processes explaining why social normative influence is amplified under uncertainty are less known. We draw on the fact that uncertainty has been shown to be associated with perceived negative emotions [42]. Building on the classical processes of social normative influence, information versus normative influence as proposed by Deutsch and Gerrard [24], we suggest that social normative influence might also have an emotion-regulating function (see also [43]). In essence, we propose that when individuals encounter anxiety and frustration resulting from decision-making under uncertainty, seeking input from others (i.e., relying on a descriptive norm heuristic) will mitigate these negative emotions.

### Emotion regulation

The term emotion regulation typically refers to individuals’ capacity to adaptively manage and respond to an emotional experience. Defined as “*a collection of implicit and explicit skills that can be used to monitor, evaluate, and modify emotional responses in accordance with one’s goals” *(Silvers, 2022), the essential feature of emotion regulation is the activation of a goal to influence the emotion trajectory [32]. For example, biting one’s lip in order to not laugh out loud at an inappropriate comment or closing one’s eyes when watching a scary movie. Although relying on emotions sometimes leads to biased decision-making, emotions can also be functional (e.g., Keltner & Gross, 1999 [38, 52]). Largely building on Lazarus (1966) concept “emotion focused coping”, research shows that emotions can be regulated for example by exercising, using drugs, or making social comparisons (see Gross, 1999 for a review). Although social comparisons can serve as a means to regulate emotions [55], current research has merely started investigating the role of emotions as motivators for following injunctive, descriptive, and personal norms [43]. To the best of our knowledge, research on the role of social norms for emotion regulation is largely lacking.

As stated above, social comparison theory proposes that people are more likely to seek social comparisons in uncertain situations [26]. More recent theoretical frameworks propose that uncertainty is unpleasant. Perceived uncertainty is likely to activate the behavioral inhibition system, which triggers negative emotions such as anxiety and fear, ultimately leading to stress [14, 16, 31, 34]. Experimental research has demonstrated that uncertainty increases negative emotional states [4], and that it can dampen positive emotions [58]. Building on research suggesting that social comparisons could mitigate such negative emotions [55], we will explore if negative emotions stemming from perceived uncertainty can be alleviated through social normative information. Learning the typical behavior of others (i.e., descriptive social norms) in an uncertain situation might mitigate the negative emotions by reducing uncertainty, after all, people often follow descriptive social norms because it is perceived as adaptive [21].

### Salience of social norms

The focus theory of normative conduct proposes that social norms are influential only when they are being focused upon, making norms cognitively salient [20]. As multiple norms might apply in a given situation, cues in the environment can serve as activators of a specific norm [19, 37]. For example, a littered environment constitutes a cue about descriptive norms, making people focus on the anti-littering norm, which has been shown to both activate normative considerations and affect littering (e.g., [7, 19]). Drawing on focus theory, we propose that in times of uncertainty, people will focus their attention more strongly on social norms. We therefore explore whether people perceive social norms as more salient under uncertainty.

### Overview of studies

In three experiments, we investigated whether social norms exert a more pronounced influence on decision-making under conditions of uncertainty, if norms become more salient under uncertainty, and whether exposing participants to a descriptive social norm mitigates negative emotions associated with decision-making under uncertainty. In Experiment 1, we tested the influence of a descriptive social norm after inducing uncertainty or not. Furthermore, we explored whether social normative information had an emotion-regulating effect and if uncertainty made people more focused on social norms. Experiment 2 sought to assess the robustness of Experiment 1. Finally, Experiment 3 broadened the definition of uncertainty, from complexity to risk versus ambiguity, and tested both the influence of social norms across these types of uncertainties and, once again, if descriptive social norms regulate negative emotions. All experiments were pre-registered at the Open Science Framework.

## Experiment 1

In Experiment 1, we test if uncertainty, operationally defined as complexity, would 1) increase the influence of descriptive social norms, 2) make social norms more salient, 3) decrease positive emotions and increase negative. Finally, we test if descriptive social norms have an emotion-regulating function.

## Method

### Design

Experiment 1 was a decision-making task assessing participants’ choice between two bottles of juice in a 2 (Uncertainty: yes vs. no) × 2 (Descriptive norm: yes vs no) between-subjects factorial design.

### Power analysis and sample recruitment

Participants were recruited through Prolific Academic and were compensated with £0.45 for completing the survey, which took approximately two minutes. All participants were informed that their participation was voluntary, anonymous, and that they had the right to withdraw at any time. All participants included in the final sample gave their consent. The power calculation was based on a similar study assessing social normative influence in an online consumer decision-making task, reporting an effect size of *w* = 0.15 [9]. Using G*Power, our power analysis indicated that 798 participants were needed for sufficient power (α = 0.05, 1-$\beta$ = 0.80). We recruited 850 participants to account for potential eliminations due to failing the attention check. 64 participants failed the attention check, implying insufficient power. We therefore recruited additional participants, ending up with a final sample of 837 participants (M_age_ = 39.3, 52% male, 46.5% female, and 1.6% preferred not to say, transgender or other) located in the United States of America.

### Materials

As a manipulation check assessing perceived uncertainty (operationalized as complexity), we used a single-item measure of complexity drawing on van Dijk and Zeelenberg (2003): “How complex do you find it choosing between the two bottles?” Responses were recorded using a 7-point scale ranging from 1 (Extremely complex) to 7 (Not complex at all).

To test emotion regulation, we measured emotions by asking participants “When thinking about which bottle to choose, how do you feel?”, using a 7-point scale ranging from 1 (Not at all) to 7 (Very much), including the emotions anxious, frustrated, confused, happy, excited, and interested, which were presented in random order [42]. Cronbach’s alpha showed acceptable internal reliability both for items measuring negative emotions (α = 0.76) and positive emotions (α = 0.84). Hence, we constructed index variables for both negative and positive emotions.

Participants in the norm conditions answered four questions measuring the salience of injunctive and descriptive norms using 7-point scales. The two items measuring a descriptive norm were: “Seeing what most others chose was useful information for me” 1 (Totally disagree) to 7 (Totally agree), “Going against what most others chose is…” 1 (Absolutely not a good idea) to 7 (Absolutely a good idea) (reverse coded), and the two items measuring an injunctive norm were: “How do you think others would react if you choose the same bottle as them” 1 (Strongly disapprove) to 7 (Strongly approve), “How do you think others would react if you did not choose the same bottle as them” 1 (Strongly disapprove) to 7 (Strongly approve) (reverse-coded). Reliability analyses did not reach acceptable levels for creating index variables for either descriptive norms (α = 0.37) or injunctive norms (α = 0.51).

As an attention check, participants were asked to indicate the color of the liquid in the bottles (red, orange/yellow, transparent, brown). Finally, participants answered two demographic items (gender and age), and were debriefed (see also Appendix B for information about the pilot study).

### Procedure

The recruitment text was presented to participants both on Prolific Academic and on the first page of the Qualtrics survey, explaining that the study focused on product preferences. After reading the introductory text, participants proceeded to the first question, where they provided their consent to participate in the study.

In all conditions, participants were asked to choose between two bottles of juice (see stimulus materials in the supplementary materials). The only difference between the bottles was the fabricated numbers 8 or 9, which were falsely attributed to the ASTM International Resin Identification Coding System (which actually ranges from 1 to 7). Participants were randomly assigned to one of the conditions in the 2 × 2 design, inducing uncertainty or not, and inducing a descriptive norm or not. The descriptive norm was induced by showing participants a picture of one of the bottles, including the text: “For your information: Most others in a previous study chose [bottle]”. In line with the definition of uncertainty, “conscious awareness of ignorance”, we set up a condition in which participants would likely perceive a severe lack of knowledge, hence perceiving it as complex as one dimension of perceived uncertainty. Thus, we expected that participants would feel what appeared to be a simple choice as a complex task. More specifically, participants in the induced complexity condition received fictive technical information about the types of plastics in the two bottles, aimed to make them perceive that they lacked knowledge about the two choice alternatives (see Appendix A). In the control condition, participants were simply asked to choose between the two bottles without any further information. If successfully inducing uncertainty, participants in the induced uncertainty condition should experience the task as more complex and perceive stronger negative emotions compared to participants in the control condition. After being presented with the bottles, participants in all conditions answered items measuring uncertainty, emotions, salience of descriptive and injunctive norms, and were asked to make a choice between the two bottles.

To create a sense of accountability for their choice (across all groups), participants were asked to make their choice between the two bottles after being informed that they will join a group. Specifically, they were instructed as follows:"Next you will make your choice and then you will join a group. Please indicate your preferred action once you are part of the group"and were asked to respond with either"I want to show my choice to the group and provide a rationale for it"or"I want to show my choice to the group."Importantly, there was no division into groups and the measurement was not included in any analysis.

### Hypotheses and research questions

More participants in the norm condition will conform to the descriptive norm (i.e., choose the “Polymethyl Carbonate” bottle) compared to participants in the control condition (H1).

Conformity to the descriptive norm will be stronger in the induced uncertain choice condition compared to the control condition (H2).

We will explore if uncertainty leads to weaker positive and stronger negative emotions [42], and if adding a descriptive social norm will increase positive emotions and decrease negative emotions (Schaumberg & Skowronek 52) (RQ1). We will explore whether injunctive and descriptive norms are more salient in a highly uncertain compared to a less uncertain choice situation (RQ2).

## Results

We regressed perceived complexity on the experimental conditions uncertainty and descriptive norm. The full model was statistically significant (*F* (2, 834) = 238.1, *p* < .001, *R*^2^_adj_ = .36). First, we obtained a main effect of uncertainty (*β* = 2.55, SE = 0.12, *p* < 0.001), indicating that participants in the induced uncertainty condition perceived the task as more complex than those in the control condition. Second, we found a non-predicted main effect of descriptive norm (*β* = −0.47, SE = 0.12, *p* < .001), showing that participants who received the descriptive norm rated the task as less complex than those who did not receive the descriptive norm.

### Choice

**Fig. 1:**
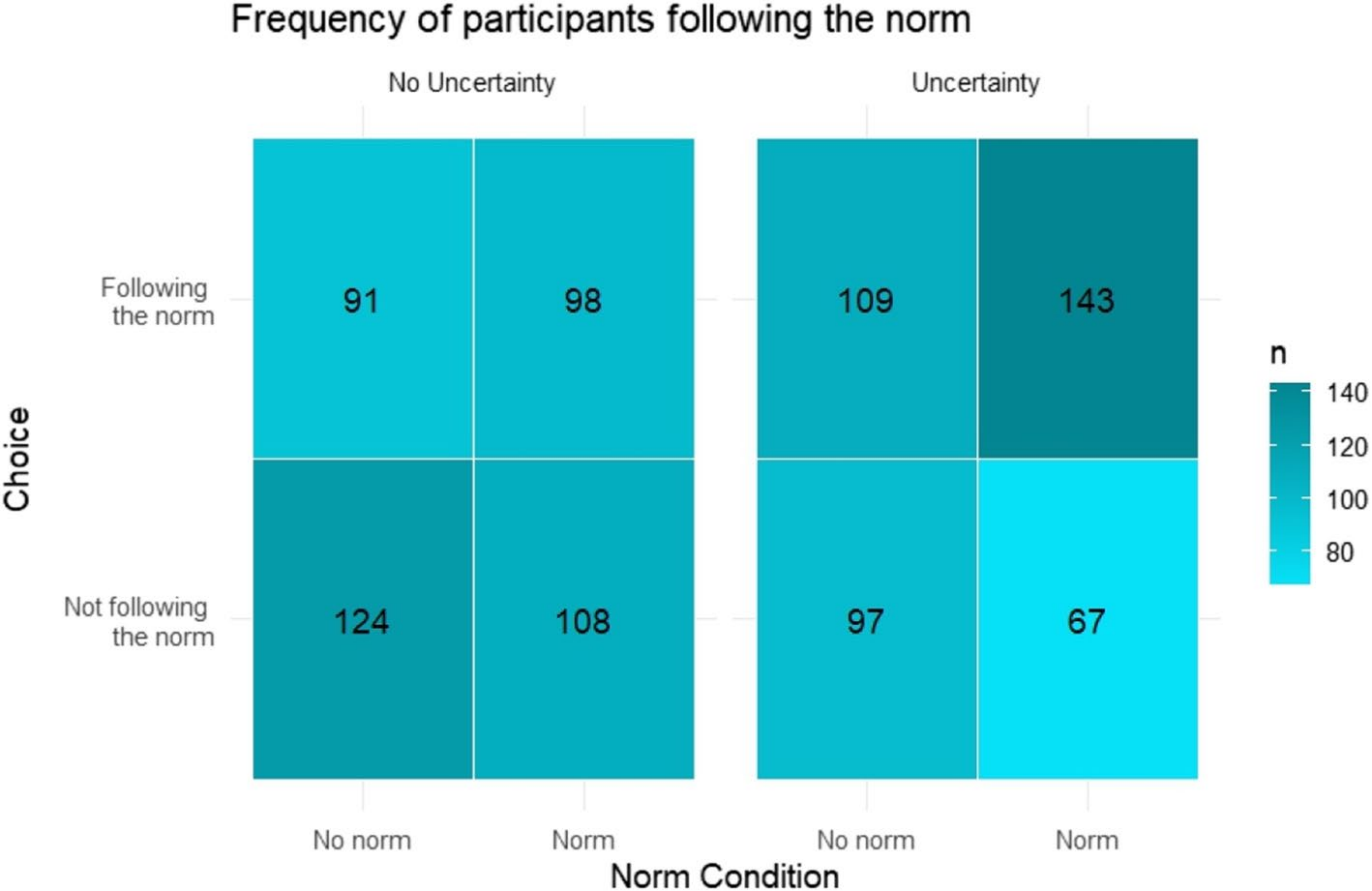
Frequency of participants following the descriptive social norm, as stated by the numerical value in each cell, across the experimental conditions in Experiment 1

Planned comparisons chi-squared analyses confirmed hypothesis 1, suggesting that the descriptive social norm influenced participants' choice (*χ2* (1, *N* = 837) = 8.71, *p* = .003, ø = 0.10). 57.9% of the participants followed the norm by choosing the “Polymethyl Carbonate” in the norm condition, while 47.5% made that choice in the control condition (i.e., no norm). Moreover, supporting hypothesis 2, the influence of social norms was statistically significant in the induced uncertainty condition (*χ2* (1, *N* = 416) = 9.41, *p* = .002, ø = 0.16), but was substantially weaker and not statistically significant in the control condition (*χ2* (1, *N* = 421) = 0.97, *p* = 0.33, ø = 0.05). A logistic regression confirmed a statistically significant norm × uncertainty interaction (*β* = 0.86, SE = 0.17, *p* < .001, see Fig. 1). This suggests that the influence of the descriptive social norm on choice was indeed moderated by uncertainty level.

### Emotion regulation

**Fig. 2:**
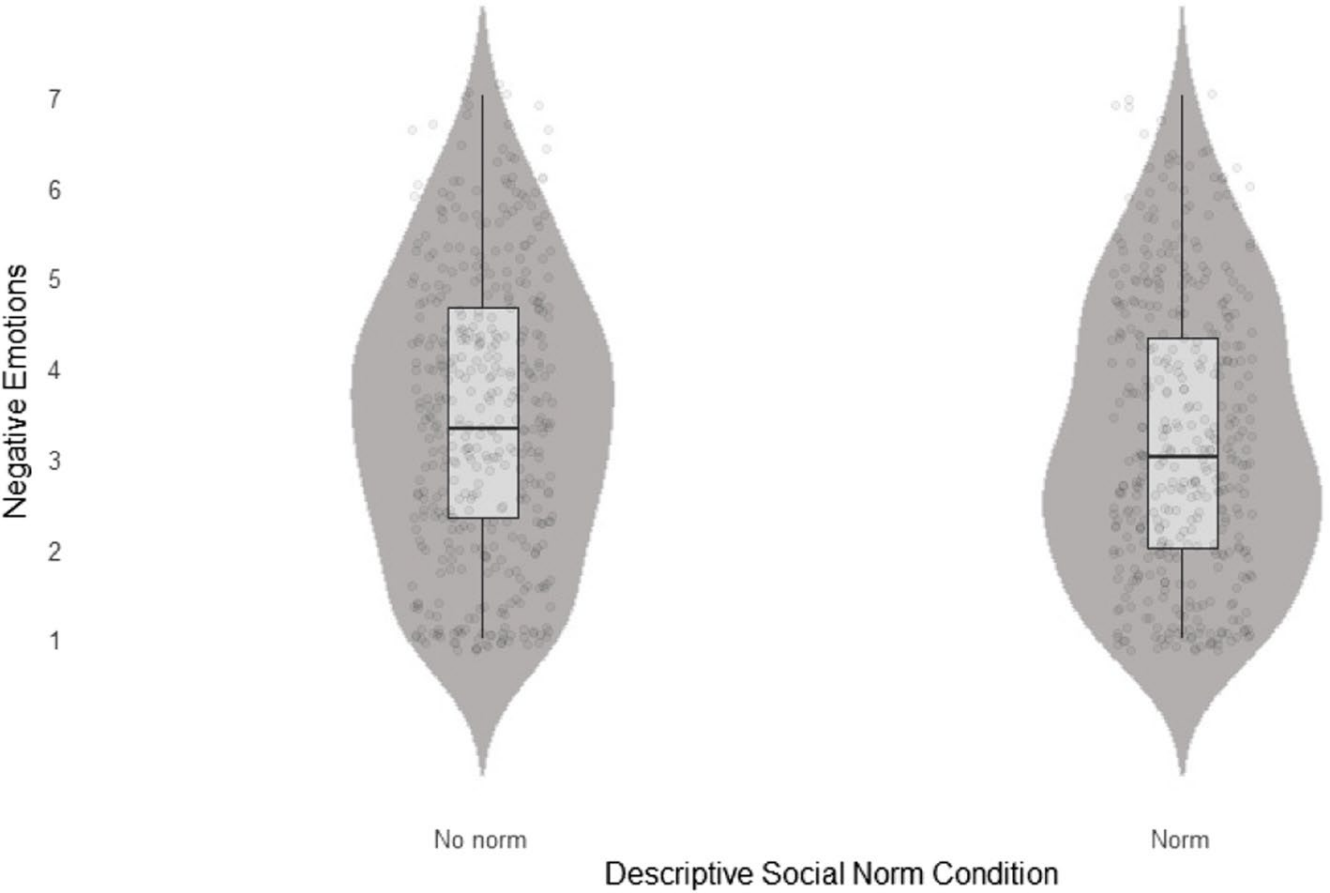
The main effect of the descriptive norm in mitigating negative emotions in Experiment 1

We regressed negative and positive emotions on uncertainty and the descriptive norm × uncertainty interaction. For positive emotions, the full model was not statistically significant (*F*(2, 834) = 0.84, *p* = .43, *R*^2^_adj_ < .001) and none of the predictors were statistically significant (uncertainty: *β* = −0.06, SE = 0.12, *p* = .61, uncertainty × norm: *β* = 0.18, SE = 0.14, *p* = 0.21). For negative emotions, however, the full model was statistically significant (*F* (2, 834) = 53.31, *p* < .001, *R*^2^_adj_ = .11). First, we obtained a substantial main effect of uncertainty (*β* = 1.16, SE = 0.13, *p* < .001), indicating that participants in the induced uncertainty condition perceived stronger negative emotions than those in the control condition. Second, we found a non-significant pattern of noteworthy effect size, indicating that participants in the descriptive norm condition perceived lower negative emotions (*β* = −0.23, SE = 0.14, *p* = .11, see Fig. 2). This finding should, however, be interpreted with caution and must be replicated to gain further clarity on whether descriptive social norms have an emotion-regulating effect or not.

Due to questionable reliability, we explored the potential influence of salience using four Bonferroni-corrected t-tests. Results showed that participants who received the uncertainty manipulation reported “seeing what most other chose” as more “useful information” (M = 4.36, SD = 1.90, *n* = 210) than those who did not receive it (M = 3.47, SD = 1.97, *n* = 206, *t*(414) = 4.67, *p* < 0.001, *d* = 0.46). No statistically significant differences (*p* < .0125 after Bonferroni correction) were observed for the items assessing expected (dis)approval of others if “…you chose the same bottle as them” (*t*(414) = 2.29, *p* = 0.02, *d* = 0.23), “Going against what most other chose is…” (*t*(414) = 1.08, *p* = .28, *d* = 0.10), and perceived (dis)approval if not choosing the same bottle as others (*t*(414) = 1.29, *p* = .20, *d* = 0.13).

## Discussion

Experiment 1 suggests that inducing uncertainty, operationalized as complexity, improved the influential power of descriptive social norms. These findings resonate with meta-analytic evidence (e.g., [12, 27]). After providing participants with complex information regarding a choice (i.e., aiming to induce uncertainty), participants were more strongly influenced by the social norms manipulation when making the choice between two plastic bottles. In seeking to advance past research, we explored psychological processes of normative influence under uncertainty. For positive emotions, the model explained close to no variance at all. For negative emotions, however, results first showed that induced uncertainty elevated negative emotions. Of particular importance, results provide some preliminary indications that adding a descriptive norm to an uncertain choice task mitigated negative emotions. The results were, however, not statistically significant and must be replicated to gain further clarity about the relationship between social norms and emotion regulation for negative emotions. The non-significant result might be due to Experiment 1 being slightly underpowered or that the induced descriptive norm was too weak to obtain a statistically significant effect. Yet, the descriptive results provide an indication, adding to the established explanations for why people conform to descriptive social norms (i.e., mimicking adaptive behaviors), by suggesting that social norms might also serve as a strategy to regulate negative anticipatory emotions. Importantly, these findings must be interpreted with caution. In gaining more confidence, we put the role of emotion regulation to test by seeking to replicate it in Experiment 2.

Building on the focus theory of normative conduct [20], we tested the plausibility that social norms will become more salient in uncertain contexts. Results provided mixed support for this proposition. Participants rated the descriptive norms as more “useful” but we found no effect for the other items [20] reported that descriptive littering norms lead to more littering when making the pro-litter norms salient (i.e., via a confederate dropping a handbill). In Experiment 1, we tested induced uncertainty as a potential method for making the norms salient. We reasoned that strengthened conformity under uncertainty might be partially explained by increased salience of the norm. Overall, results did not support this expectation. First, it should be mentioned that the strength of our experimental manipulation was probably much weaker than the one [20] developed. Second, we did not assess the behavioral consequences of salience but developed four items aimed to assess salience. The items have not been validated. Hence, the construct validity is not established. We will, however, replicate the test of salience in Experiment 2 to gain more confidence in the null findings.

Experiment 1 was limited in at least three regards. First, uncertainty was operationalized as complexity and validated by one manipulation check item. Therefore, in Experiment 2, we aim to use a broader manipulation-check of uncertainty, assessing if this experimental manipulation affected task complexity, task difficulty, feelings of uncertainty, and confidence. Second, Experiment 1 was also limited by the fact that we obtained an unexpected main effect of uncertainty on choice, confounding both the main effect of social norms and the interaction. Third, the operational definition of uncertainty as complexity might be confounded with cognitive load, such that participants who received the uncertainty inducing stimuli may have experience greater cognitive load than those not receiving it. This is important because heuristics are more influential under cognitive load (e.g., Fredrick & Kahneman, 2007, see also [59]), and therefore, people may be more prone to follow the descriptive norm compared to the control condition. This is potentially a fundamental limitation of the current study, questioning the internal validity. If the experimental manipulation provided participants with complex information that induced both uncertainty and cognitive load, the experiment did not succeed in testing the effect of uncertainty in isolation. In assessing the potential risk for cognitive load, we will explore the time participants take to answer items on emotions in Experiment 2, as this was followed directly after induced uncertainty. If deprived by cognitive load, we expect participants to spend less time on these items. Finally, Experiment 2 will also advance Experiment 1 by asking participants to avoid (rather than choose between) one of two pesticides, thus introducing potential danger to make the choice less trivial.

## Experiment 2

The aim of Experiment 2 is to assess the robustness of two key findings from Experiment 1, that 1) induced uncertainty amplifies the effect of descriptive social norms, 2) uncertainty increases negative emotions, which is in turn alleviated by the addition of descriptive social normative information, suggesting that social norms have an emotion-regulating effect. In addition, in Experiment 2, we will once again assess if social norms are more salient under highly uncertain compared to less uncertain situations.

In Experiment 2, we advanced Experiment 1 by operationalizing the descriptive norm as a “don’t-norm”, implying that the norm will be presented as what others avoid, rather than what they choose (i.e., “do-norms”). Past research has shown that don’t-norms are more influential than do-norms, plausibly because people generally have a stronger tendency to avoid than approach Baumeister et al., [6, 8]. By using a more influential operationalization of the descriptive norm, we expect less noise in assessing the role of uncertainty in social normative influence. Second, in Experiment 1, participants in the induced uncertainty conditions reported higher negative emotions compared to those in the control condition. Descriptive results suggested that negative emotions tended to be mitigated by adding a social norm. No statistically significant effects of positive emotions were obtained. In Experiment 2, we will seek to replicate the effect of negative emotions only, excluding positive emotions because of the lack of effect in Experiment 1 [42, 48]. Third, using the same salience measures as Experiment 1, we aim to assess if uncertainty makes the descriptive norm more cognitively salient. Finally, in Experiment 2, we aim to test a broader manipulation-check of induced uncertainty, including task complexity, task difficulty, feelings of uncertainty, and confidence.

## Method

### Design

We will assess the hypotheses using a 2 (Uncertainty: yes vs no) × 2 (Norm: yes vs no) between-subjects factorial design on ratings of ambiguity, negative emotions, and salience of social norms.

### Power analysis and sample recruitment

Participants were recruited through Prolific Academic with the pre-screening conditions of people living in the United States, and to not previously have taken the surveys connected to the project. Participants were paid £0.45, and the survey took on average 3 min to complete. All participants were informed that their participation was voluntary, anonymous, and that they had the right to end their participation at any time. All included participants gave their consent. The power calculation was based on the effect of social norms in highly uncertain situations, as obtained in Experiment 1 (w = 0.14). Using G*Power, we decided to recruit at least 802 participants to reach sufficient power (α = 0.05, 1-*β* = 0.8). Consequently, we recruited 900 participants to account for potential exclusions due to missed attention-check. As 25% of the participants failed the attention check, we recruited 200 additional participants leaving 847 (M_age_ = 39.9, 50.9% male, 47% female, and 2.1% preferred not to say, transgender or other) participants for the final sample.

### Measures

Measures of choice were the same as Experiment 1, except that participants were instructed to “avoid” rather than “choose” one of the two bottles. Negative emotions were assessed using the same items as in Experiment 1. Cronbach’s alpha showed acceptable reliability for items measuring negative emotions (α = 0.73), we, therefore, analyzed emotions using index variable. Measures of salience were identical to Experiment 1, including four items assessing both descriptive and injunctive norms. Cronbach’s alpha reliability test did not reach acceptable levels for creating index variables for either descriptive norms (α = 0.61) or injunctive norms (α = 0.54).. In broadening the measures of uncertainty, to include both complexity and uncertainty, participants were presented with four questions, all measured on 7-point Likert scales: “How complex do you find the task of choosing between the two pesticides?” from 1 (not complex at all) to 7 (Extremely complex), “How difficult do you find the task of choosing between the two pesticides?” from 1 (Not difficult at all) to 7 (Extremely difficult), “How (un)certain do you feel when choosing between the two pesticides?” from 1 (extremely uncertain) to 7 (extremely certain), “How (un)confident do you feel when choosing between the two pesticides?” from 1 (extremely unconfident) to 7 (extremely confident). Measures of uncertainty showed acceptable Cronbach’s alpha reliability (α = 0.76), we therefore created one index variable for the uncertainty-measures.

Participants were asked what color the cap of the pesticide bottles was, with four options (red, black, green, or blue). As in Experiment 1, participants finally answered two demographic items (gender and age) and were briefed. See Appendix for pilot studies.

### Procedure

We used the same procedure and design as in Experiment 1, apart from using new stimulus materials and an alternative framing of the decision task. In Experiment 2, participants were asked to choose which of the two bottles of pesticides they wanted to avoid. Like Experiment 1, the bottles were the same apart from their names. In the two norm conditions, there was also a picture of one of the bottles with the descriptive norm: “Most others in a previous study chose to avoid this pesticide”. In the induced uncertainty conditions, there was fictional technical information about the two different, made-up types of pesticides, whereas no such information was present in the control conditions (see Appendix).

### Hypotheses

More participants in the norm condition will choose the pesticide “Bromochlorophenoxylamine” (i.e., conform to the descriptive norm) than in the control condition (H1).

Conformity to the descriptive norm will be stronger in the induced uncertainty condition than in the control condition (H2).

Participants in the induced uncertainty conditions will report stronger negative emotions than those in the control conditions (H3a). Participants exposed to the descriptive norm will report weaker negative emotions than those not exposed to the descriptive norm (H3b).

Participants in the induced uncertainty condition will show stronger activation of the descriptive norm than those in the control condition (H4a).

Participants in the induced uncertainty condition will show stronger activation of the injunctive norm than those in the control condition (H4b).

Furthermore, we will assess participants’ perception of uncertainty in a manipulation check and assess if displaying a descriptive social norm leads people to perceive the choice situation as less uncertain.

## Results

First, in exploring cognitive load as a potential confounder, we regressed the time spent answering emotions items on descriptive norm, uncertainty, the descriptive norm × uncertainty interaction. The full model was not statistically significant (*F*(3, 843) = 0.23, *p* = .87, *R*^2^_adj_ < .001). None of the main effects (norm: *β* = 1.71, SE = 2.6, *p* = .51, uncertainty: *β* = 1.91, SE = 2.58, *p* = .46) nor the interaction (*β* = −2.71, SE = 3.68, *p* = .46) were statistically significant. Second, in analyzing the manipulation check, we regressed the index variable of perceived uncertainty on descriptive norm, uncertainty, and the descriptive norm × uncertainty interaction. The full model was statistically significant (*F*(3, 843) = 3.72, *p* = .01, *R*^2^ = .01). Confirming the experimental manipulations, we obtained a statistically significant main effect of uncertainty (*β* = 0.40, SE = 0.13, *p* = 0.003). We found no main effect of norm (*β* = 0.07, SE = 0.13, *p* = 0.57), however, a significant interaction (*β* = −0.43, SE = 0.19, *p* = .02). Taken together, these results suggest no evidence for differences in cognitive load between participants in the experimental and control condition, while the experimental manipulation successfully induced perceived uncertainty. Once again, we observed that among participants induced by uncertainty, those who also received a descriptive social norm perceived the task as less uncertain than those who did not.

### Choice

**Fig. 3:**
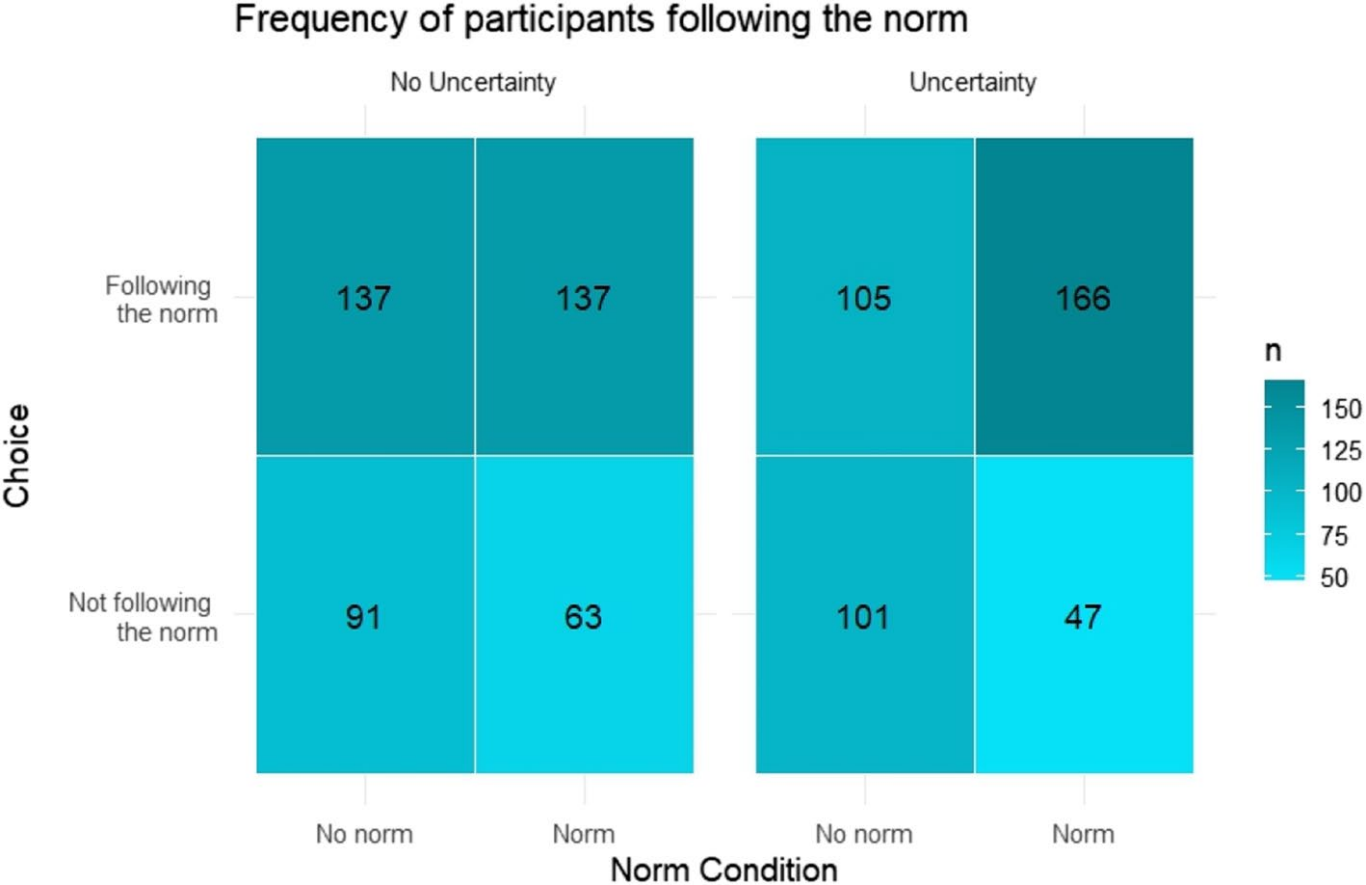
Frequency of participants following the descriptive social norm, as stated by the numerical value in each cell, across the experimental conditions in Experiment 2

Planned comparisons using chi-squared analyses confirmed Hypothesis 1, that choice was affected by the descriptive social norm (*χ2* (1, *n* = 847) = 27.82, *p* < .001, ø = 0.18). Moreover, confirming hypothesis 2, the influence of social norms was statistically significant under uncertainty (*χ2* (1, *n* = 419) = 32.16, *p* < .001, ø = 0.28), but substantially weaker and not statistically significant in the control condition (*χ2* (1, *n* = 428) = 2.92, *p* = .09, ø = 0.09). To test the interaction hypothesis, we ran a binary logistic regression showing a statistically significant norm × uncertainty interaction (*β* = 0.87, SE = 0.18, *p* < .001, see Fig. 3). These results replicate the findings from Experiment 1 and confirm that the impact of descriptive norms on choice behavior is moderated by uncertainty.

### Negative emotions

**Fig. 4:**
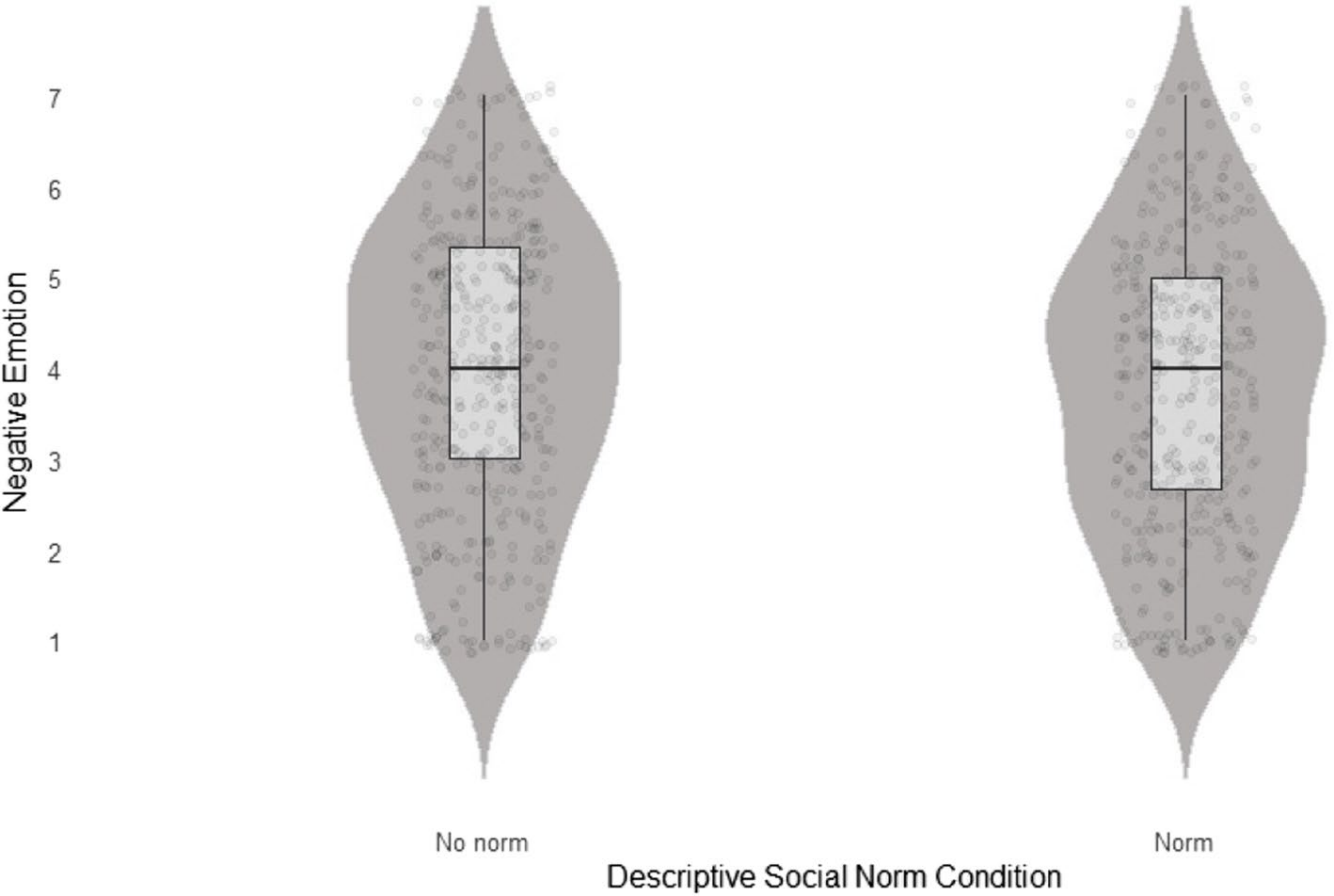
The effect of the descriptive norm in mitigating negative emotions under high uncertainty in Experiment 2

We regressed negative emotions on uncertainty and the descriptive norm × uncertainty interaction. The full model was statistically significant (*F* (2, 844) = 4.91, *p* = .008, *R*^2^_adj_ = .009). First, we obtained a main effect of inducing uncertainty (*β* = 0.40, SE = 0.14, *p* = .003), showing that participants in the induced uncertainty condition reported stronger negative emotions than those in the control condition. Second, we found a statistically significant interaction (*β* = −0.40, SE = 0.16, *p* = .01). These results replicate Experiment 1 in showing that inducing uncertainty elevated participants' negative emotions. Importantly, when presented with a descriptive norm, these negative emotions were mitigated. Hence, we replicated the descriptive trend as obtained in Experiment 1, now showing a statistically significant interaction between negative emotions and the descriptive social norm. These results suggest that the descriptive social norm served an emotion-regulating function (Fig. 4).

### Norm salience

In assessing the effect of salience, we analyzed all four items separately using Bonferroni-corrected t-tests (*p* < .012). For both injunctive norms, we obtained no statistically significant effect (*t*(411) = 0.90, *p* = .36, *d* = 0.09, and *t*(411) = 1.52, *p* = 0.13, *d* = 0.15, for the first and second measures, respectively). For descriptive norms, we found no statistically significant effects for either “Seeing what most others chose was useful information for me” (*t*(411) = 1.80, *p* = .07, *d* = 0.18) or “Going against what most others chose is…” (*t*(411) = 2.03, *p* = .04, *d* = 0.20), although the results were descriptively in the predicted direction. Taken together, these results do not support hypotheses 4a and 4b.

## Discussion

The results of Experiment 2 largely replicate the findings from Experiment 1. First, the manipulation check confirmed that participants in the induced uncertainty conditions experienced stronger uncertainty, using a broader definition than the one based solely on complexity as used in Experiment 1. Relatedly, assessing cognitive load by the time it took participants to answer the items on emotions, results showed no statistically significant effect, indicating that participants in the induced uncertainty condition were not more cognitively deprived than those in the control condition.

Second, both Experiments 1 and 2 restricted the operational definition of uncertainty as conscious awareness of ignorance, assessed by complexity in Experiment 1, and both complexity and uncertainty in Experiment 2. In broadening the definition of uncertainty, another important distinction is between risk, where probabilities are known, and ambiguity, where probabilities are unknown [25]. Therefore, in Experiment 3, we aim to explore the amplified influence of social norms under ambiguity and risk. Second, in Experiment 3, we aim to replicate the emotion-regulating function of descriptive norms. Now assessed by the two items: anxiety and frustration.

## Experiment 3

### Design

Experiment 3 will use a 2 (Uncertainty: ambiguity vs risk) × 2 (Norm: yes vs no) between-subjects factorial design on choice and ratings of negative emotions.

### Power analysis and sample recruitment

The power calculation was once again based on Experiment 1. We recruited 850 participants from the UK or USA through Prolific Academic for an estimated hourly payment of £9.

### Procedure

In Experiment 3, participants were asked to support one of two nuclear power plants (called Ringhals and Barsebäck, each depicted with a picture) that faced the risk of a meltdown. This material was chosen based on a pilot study including 150 participants, which largely displayed social normative influence in four scenarios of which the Ringhals/Barsebäck scenario was the most evenly distributed in the control condition in terms of choice (see Appendix D). Consistent with Experiments 1 and 2, participants were randomly assigned to a norm or a no-norm condition. In the norm condition, the descriptive social norm was induced by displaying the following text: “For your information. Most participants choose to support Ringhals” together with a picture of that power plant. In advancing Experiments 1 and 2, uncertainty was operationalized and assessed as either ambiguity or risk. Participants assigned to the risk condition learned that: “The risk of meltdown in the two nuclear power plants is 50–50, meaning that both nuclear power plants have an equal probability of being affected.”*.* Participants in the ambiguous condition learned that: “The risk of meltdown in the two nuclear power plants is unknown, indicating that the probability for each of the nuclear power plants is also uncertain.”

### Choice

Measures of choice were the same as Experiment 1, apart from asking participants to “support” rather than “choose” one of the two power plants.

### Negative emotions

Measures of negative emotions included two items (*anxiety* and *frustration*) both assessed after the choice by asking participants “How did you feel when making the decision” assessed on a 7-point scale semantically anchored at 1 “not at all” and 7 “very much”. Cronbach’s alpha showed acceptable reliability for items measuring negative emotions (α = 0.80), we, therefore, analyzed emotions using index variables for negative emotions.

### Attention check

As an attention check, participants were asked to state which option most others prefer.

## Demographics

As in Experiment 1, participants finally answered two demographic items (gender and age) and were briefed.

### Hypotheses

Hypothesis 1 (H1): More participants in the norm condition will choose the “Ringhals” power plant (i.e., conforming to the descriptive norm) than in the control condition.

Hypothesis 2 (H2): Conformity to the descriptive norm will be stronger in the ambiguous condition compared to the risk condition.

Hypothesis 3 (H3): Based on the results from Experiment 2, we hypothesize an interaction in the ambiguity condition, showing that the presence of a norm will weaken negative emotions compared to those who do not receive a norm.

## Results

### Attention-check

Twelve participants failed the attention check, leaving 838 participants to the final analysis (M_age_ = 40.8, 39.4% male, 59.5% female, and 1.1% preferred not to say, transgender or other).

### Choice

**Fig. 5:**
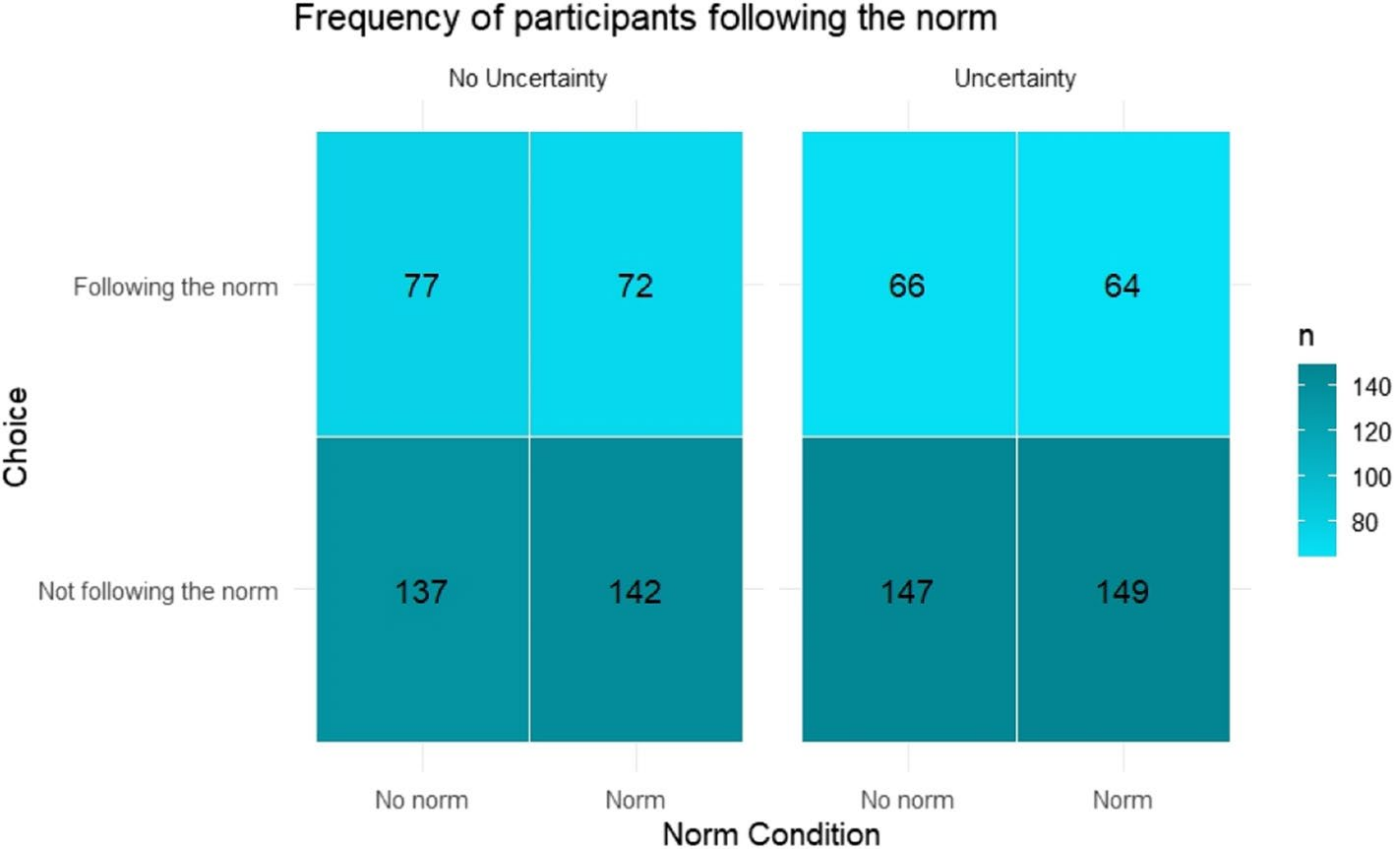
Frequency of participants following the descriptive social norm across the experimental conditions in Experiment 3

A planned comparisons chi-squared test rejected hypothesis 1, that choice was affected by the descriptive social norm (*χ2* (1, *n* = 854) = 0.19, *p* = .66, ø = −0.02). Furthermore, hypothesis 2 was also rejected, as descriptive social norms did not include choice neither under risk (*χ2* (1, *n* = 426) = 0.01, *p* = .92, ø = −0.01), or ambiguity (*χ2* (1, *n* = 428) = 0.16, *p* = .68, ø = −0.03). These results were validated in a binary logistic regression showing no interaction (*β* = −0.16, SE = 0.17, *p* = .35, see Fig. 5).

### Negative emotions

**Fig. 6:**
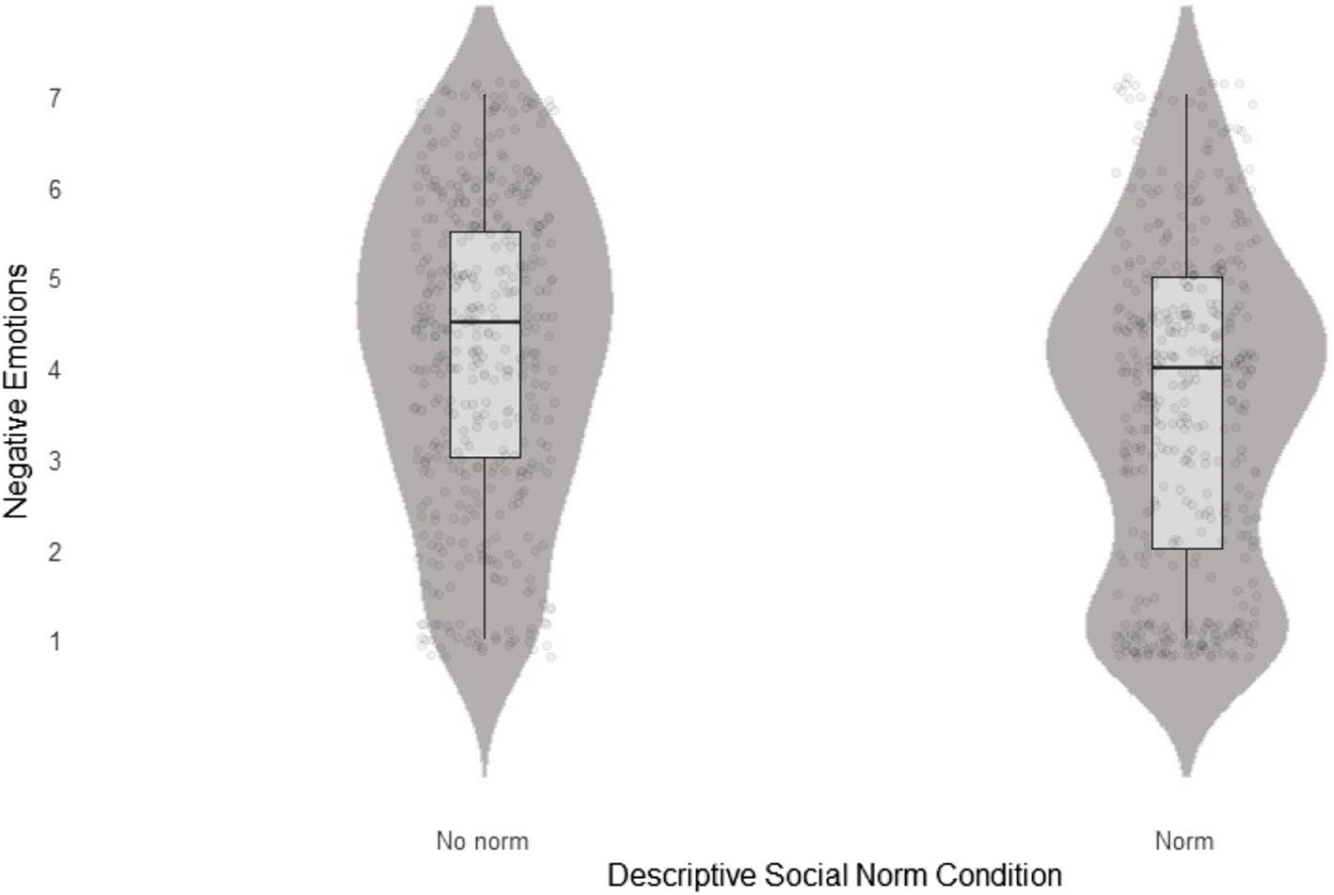
The main effect of the descriptive norm in alleviating negative emotions in Experiment 3

We regressed negative emotions on uncertainty, the descriptive norm, and the norm × uncertainty interaction. The full model was statistically significant (*F* (3, 850) = 8.46, *p* < 0.001, *R*^2^_adj_ = 0.03). Neither the main effects of uncertainty (*β* = −0.03, SE = 0.17, *p* = 0.85) nor the interaction (*β* < −0.001, SE = 0.24, *p* = 0.99) was statistically significant, rejecting H3. For the main effect of norm, however, we replicated Experiments 1 and 2, finding a significant main effect (*β* = −0.60, SE = 0.17, *p* < 0.001) showing that participants who received the descriptive social norm reported lower negative emotions. Once again, suggesting that descriptive social norms have an emotion-regulating function (Fig. 6).

## Discussion

The results of Experiment 3 partially replicate the findings from Experiments 1 and 2. First, Experiment 3 did not support the hypothesis that social normative influence was amplified under ambiguity compared to risk. However, it should be noted that both conditions induced uncertainty in contrast to Experiments 1 and 2, comparing induced uncertainty to the control condition. Second, in line with Experiments 1 and 2, we once again obtained that adding a descriptive social norm to the choice situation alleviated negative emotions.

## General discussion

The established explanation for why social norms affect judgment and decision-making is that people are seeking or avoiding others (dis)approval and/or are using others’ behaviors as social guidelines for the correct or adaptive behaviors in a specific situation e.g., [18]. In three experiments, we tested the hypothesis that social norms are more influential under uncertainty. Results from Experiments 1 and 2 demonstrated that social norms are indeed more influential in uncertain situations. Experiments 1 and 2 induced uncertainty by adding complexity to the choice situation. Experiment 3 broadened the operational definition of uncertainty by testing if social normative influence is stronger under ambiguity than risk. Results showed no main effect of social norms on these two operational definitions of uncertainty. Importantly, this does not contradict Experiments 1 and 2 but fails to show a difference between the two types of uncertainties – risk and ambiguity. Overall, these Experiments provide experimental evidence that more participants relied on the heuristic of descriptive social norms under uncertainty, operationalized as complexity. These findings resonate with the large body of research on heuristic decision making, suggesting that heuristics are more likely to be used under uncertainty e.g., [18, 30, 57].

Delving into the decision-making tasks in our experiments, the first and second experiments involved quite trivial decisions—choosing between two plastic bottles. Although such trivial decisions could be used to assess the influence of descriptive social norms, participants’ motivation was likely low. In contrast, the third experiment involved a much more provocative task—the risk of a meltdown at two nuclear power plants. The elaboration likelihood model (Petty & Cacioppo, 1986) predicts that social influence via the peripheral route of persuasion is more likely when motivation is low. This may explain the lack of main effect for descriptive social norms in Experiment 3. Given that descriptive social norm is a type of decision-making heuristic [22], in making that decision, although fictitious, participants might have been less likely to rely on the peripheral route of persuasion. Anecdotally, one participant stated that she *“wouldn’t want to be swayed by the group…”* when making this decision. The lack of main effect in Experiment 3 is also broadly consistent with meta-analytic findings on the bystander effect, which suggest that others’ inaction is less influential in unambiguously dangerous situations (Fischer et al., 2011). Taken together, participants’ tendency to adopt the heuristic of following the norm could have been amplified in Experiments 1 and 2, where decisions were low-stakes, and diminished in Experiment 3, where the severity of the scenario may have encouraged more deliberate processing over reliance on peripheral cues.

Our experiments explored why social norms might have a greater impact when situations lack clarity. First, Experiments 1 and 2 confirmed that induced uncertainty increased negative emotions. Participants felt anxious, frustrated, and confused when deciding under uncertainty (see Appendix C). While Experiment 3 found no statistically significant difference between the two types of uncertainties, risk and ambiguity.

That descriptive social norms regulated negative emotions under uncertainty was merely a non-significant descriptive trend in Experiment 1. Importantly, this trend was replicated in Experiment 2 with statistical significance and In Experiment 3, assessing risk vs. ambiguity, we obtained a significant main effect of descriptive norm. Essentially, we obtained consistent results that descriptive social norms served as a means to regulate negative emotions, both when a decision-maker is made consciously aware of their ignorance and faces uncertainty, as known or unknown probabilities. Seeking to advance past research on the motivations for why people follow social norms e.g., [18, 43, 56], we suggest that social norm are not only followed because people want to be accurate or avoid social sanctions – it might also serve as an emotions regulator.

We also explored whether salience could potentially explain why social norms are more influential in uncertain situations. While salience is central in the focus theory of normative conduct [20], the conditions under which social norms become more or less salient are understudied within the field e.g., [21], 50]. Based on the rationale that uncertain situations lead people to focus on social norms as a means to reduce perceived uncertainty. Results for this alternative explanation were not supported, with one exception. In Experiment 1, we found that participants who made their choice in the induced uncertain situation did, in fact, report that descriptive norms were more “useful information” than those in the less uncertain situation. It should, however, be noted that we found no statistically significant effect for other measures of salience in either of the experiments and the effect of “usefulness” was not replicated in Experiment 2. Moreover, our tests of salience were limited in at least two aspects. First, the measures of injunctive and descriptive norm salience showed low internal reliability, and the validity was not verified. Second, the approach of assessing salience via the perceived strength of norms could be conceptually criticized, as salience is a matter of attention and might therefore be more or less dichotomous – attending to the norm or not. Also, the experiment might already have made the descriptive norm salient by pointing to it in both the stimulus material and by asking the questions. The disconfirmed salience hypotheses should therefore be interpreted with caution, yet we found no convincing evidence to support it.

In Experiment 1, we obtained an unexpected finding when assessing the uncertainty of choice: a main effect of descriptive norms hinted that displaying a descriptive norm made participants perceive the situation as less uncertain. Interestingly, this finding was replicated in Experiment 2, suggesting that participants perceived the situation as less uncertain when being informed about what other people chose. These data cast light on the multiple functions of descriptive norms. In coping with uncertainty, a descriptive norm shapes decision-making and regulates negative emotions, as discussed above. A plausible process explanation is that descriptive norms made the induced uncertainty appear less uncertain, reducing the negative emotions associated with making an uncertain decision.

In assessing risk, the Iowa Gambling Task Bechara et al., 1994; [15] and the Balloon Analogue Risk Task (Lejuez et al., 2022) are commonly used methods. Importantly, these methods were primarily developed to assess risky decision making, rather than inducing uncertainty. Although participants may perceive uncertainty when performing these tasks, they are more closely related to risk than complexity. Following our definition of uncertainty, as the conscious awareness of ignorance, encompassing complexity, risk, and ambiguity [1], we developed a method to*induce complexity*in Experiments 1 and 2. By adding fictive information, we aimed to induce a sense of complexity among participants, leading to perceived uncertainty. The manipulation checks confirmed that complexity was successfully induced in both experiments. One the one hand, this could be seen as a strength as we developed and validated an experimental method for inducing uncertainty. One the other hand, although it was validated with one item, the construct validity is not fully established. In Experiment 3, we broadened our definition of uncertainty to comprise both risk, where likelihoods are known, and ambiguity, where likelihoods are unknown [25, 28]. Following this definition, our operational manipulation in Experiment 3 was to display risk by providing probability estimates and uncertainty by not providing such precise estimates of the risk. This method largely aligns with Jan et al. (2011), inducing ambiguity by presenting confidence intervals (vs. point estimates in the control group). Yet, our validations of these methods are rudimentary. We therefore encourage future research to develop and validate methods to induce, rather than measure, uncertainty.

Past research has measured and induced social norms using a plethora of methods e.g., Constantino et al., [23, 33, 41]. In all Experiments, we induced norms by an explicit method, simply describing what most others chose. First, although this method has been widely used e.g., [35, 54], it is confounded with asymmetric attention. That is, adding any information about one of the choice alternatives might draw attention to that alternative and consequently influence participants'choice (see Carrasco 17 for review). Second, even if descriptive norms were validly included, our manipulation of social norms was restricted to the descriptive social norm. We encourage future research to examine whether injunctive social norms, or the combination of aligned or misaligned injunctive and descriptive norms e.g., [21, 53], also have an emotion-regulating effect.

In sum, we experimentally demonstrated that descriptive norms (induced by explicitly stating what most others choose) are more influential in shaping decision-making as situations become more uncertain (i.e., when making participant perceive the choice as complex and uncertain), while no effect was found when operationalizing uncertainty as known versus unknown probabilities. Tapping into the psychological processes, results suggest that negative emotions were mitigated when displaying a descriptive social norm. We suggest that turning to others has an emotion-regulating effect.

## Supplementary Information

Supplementary Material 1

## Data Availability

Data and materials from all three experiments are publicly available at the Open Science Framework. Data is available on OSF: https://osf.io/mztcs/?view_only = 6094ac911d664a9da0a668322d57566d.
