# Supplementary material for: The role of emotion regulation in normative influence under uncertainty
Source: BMC Psychol. 2025 Jul 4;13:731. doi: 10.1186/s40359-025-03033-z (PMC12228370; doi:10.1186/s40359-025-03033-z)
Supplement: Supplementary file 1 — Supplementary Material 1 [file 40359_2025_3033_MOESM1_ESM.docx]

**Appendix A**

Stimulus material used to induce uncertainty as used in Experiment 1.

“This bottle is made of a plastic called Polymethyl Carbonate
Polymethyl Carbonate (PMC) is a novel, synthetic polymer that is produced by the polymerization of methylene carbonate monomers. The polymerization process is catalyzed by a proprietary organocatalyst system that enables precise control over the molecular weight and dispersity of the resulting polymer. PMC is a highly versatile plastic with a wide range of physical and chemical properties that can be tailored to meet specific application requirements. PMC is composed of repeating units of methylene carbonate, which is a cyclic carbonate ester with the chemical formula C3H4O3. The polymerization process results in a linear, thermoplastic polymer with a molecular weight range of 30,000 to 300,000 g/mol. The polymer has a glass transition temperature (Tg) of approximately 60°C, which makes it suitable for use in a wide range of temperatures.”

“This bottle is made of a plastic called Polysulfone Methacrylate
Polysulfone Methacrylate (PSMA) is a novel, synthetic polymer that is produced by the copolymerization of sulfone monomers and methacrylate monomers. The copolymerization process is catalyzed by a proprietary catalyst system that enables precise control over the monomer ratios and molecular weight distribution of the resulting polymer. PSMA is a highly versatile plastic with a wide range of physical and chemical properties that can be tailored to meet specific application requirements. PSMA is composed of repeating units of sulfone and methacrylate monomers, which result in a linear, thermoplastic copolymer with a molecular weight range of 50,000 to 500,000 g/mol. The polymer has a glass transition temperature (Tg) of approximately 190°C, which makes it suitable for use in high-temperature applications.”

Note, the plastics do not exist in reality and the facts about them are false. They were made up for the experiment to create the induced unceratainty situation.

Stimulus material used to induce uncertainty as used in Experiment 2.

Bottle 1

“Mode of Action: Bromochlorophenoxylamine is an organochlorine pesticide that exerts its activity by irreversibly inhibiting the acetylcholinesterase enzyme in pests. By disrupting the hydrolysis of acetylcholine, the neurotransmitter responsible for transmitting nerve impulses, it impairs the normal functioning of the nervous system, leading to paralysis and subsequent mortality in target organisms.

Persistence: Bromochlorophenoxylamine demonstrates a high degree of environmental persistence, characterized by a half-life of approximately 120 days. Its stability in soil, water, and plants for an extended duration raises concerns regarding the potential accumulation and bioavailability in non-target organisms and ecosystems.

Toxicity: Bromochlorophenoxylamine is classified as highly toxic to mammals, exerting pronounced adverse effects on various physiological systems, including the nervous, respiratory, and reproductive systems. Prolonged exposure or ingestion of this pesticide has been associated with the development of chronic health issues, encompassing developmental abnormalities, carcinogenicity, and endocrine disruption.”

Bottle 2

“Mode of Action: Fluorochlorophenoxylamine, a carbamate pesticide, acts as a reversible inhibitor of cholinesterase enzymes in pests, thereby impeding proper nervous system functioning. By interfering with the hydrolysis of acetylcholine, a crucial neurotransmitter, it leads to the accumulation of acetylcholine at nerve synapses leading to paralysis, and subsequent mortality in target organisms.

Persistence: Fluorochlorophenoxylamine exhibits a moderate level of environmental persistence, with a half-life of approximately 120 days. Despite partial degradation due to factors like sunlight exposure and microbial activity, it remains persistently present in the ecosystem, posing ongoing risks to soil and water contamination.

Toxicity: Fluorochlorophenoxylamine poses high toxicity risks to mammals, eliciting acute poisoning symptoms, including nausea, vomiting, excessive sweating, and respiratory distress. Prolonged exposure or ingestion of this pesticide has been associated with the development of chronic health effects, encompassing neurotoxicity, hepatic and renal damage, and disruption of the endocrine system.”

Note, the pesticides do not exist in reality and the facts about them are false. They were made up for the experiment to create the highly ambiguous situation.

**Appendix B**

*Pilot study for Experiment 1.* To assess the distribution of choice, we recruited 90 participants from Prolific to a pilot study. Participants were randomized into either a induced uncertainty condition or a control condition and asked to choose between the two bottles of orange juice. The bottles were marked with a circular or a cross-like symbol, and both conditions included descriptive norm information displaying that “most other in a previous study” choose the bottle with the cross-like symbol. Further, we tested an injunctive norm question to test the baseline of which of the two options participants chose. Results indicated that the baseline distribution was skewed as 32% follow the norm in the control condition, and 43% in the induced uncertainty condition. Although indicative of the prediction that social norms are more influential under uncertainty, conformity was lower than 50% in both norm conditions, indicating a preference for the bottle with a circle-like symbol. Based on these results we exchanged the symbols for Experiment 1 for a plastic resin symbol including numbers 8 and 9 as they do not exist (7 is the final as it stands today).

Pilot study 1 for Experiment 2: To assess the distribution of choice, we recruited 90 participants from Prolific to a pilot study. Participants were randomized into either a induced uncertainty or a control condition and asked to participants which pesticide they would avoid, choosing between to identical bottles namned “Methyloxychloronitrophenol” and “Chloronitrophenoxyphenol”. Results indicated that the baseline distribution was skewed as 34% choose to avoid the “Methyloxychloronitrophenol” in the low ambiguity condition, and 36% in the induced uncertainty condition. We, therefore, ran a second pilot study to develop stimulus material with a baseline as close to a 50-50 distribution as possible.

Pilot study 2 for Experiment 2: In the second pilot study, we recruited 20 participants using a convenience sampling, and asked them to choose which of the two bottles in ten different pairs of pesticides they would avoid, on a scale ranging from -3 to 3. This time the name Methyloxychloronitrophenol was not included. We chose the pair where most of the participants had answered 0, and which also had the lowest standard deviation (“Bromochlorophenoxylamine” and “Fluorochlorophenoxylamine”) to be further tested in a third pilot study.

Pilot study 3 for Experiment 2: To test both the distribution of choice and extended measurement of uncertainty , we ran a pilot study recruiting 90 participants from Prolific. Participants were randomly assigned to one of three conditions (induced uncertainty, induced uncertainty + norm, or a control condition) and asked to choose to avoid the “Bromochlorophenoxylamine” or the “Fluorochlorophenoxylamine” pesticide. In the norm condition, participants were informed that “most others in a previous study choose to avoid this pesticide” followed by a picture of the “Bromochlorophenoxylamine”. First, Cronbach's alpha showed acceptable reliability for the four items measuring uncertainty (α =. 73, n = 90). Second, the choice was descriptively less skewed compared to pilot study 1, yet not equally distributed as the “Bromochlorophenoxylamine” pesticide was chosen by 60% of participants in the control condition, 67% of participants in the induced uncertainty condition and by 60% of the participants in the induced uncertainty plus descriptive norm condition. These results are not in line with our hypothesis. However, as the sample size was relatively small, the effect could be explained by random noise in the data. As these results were our most informed choice at this point, we choose to use the stimulus material in Experiment 2.

**Appendix C**

Consistent with the pre-registration, we ran a 2 (Uncertainty: yes vs no) × 2 (Norm: yes vs no) independent MANOVA on rating of the three items measuring negative emotions with the data from Experiment 1. Results showed statistically significant main effects of uncertainty for all three emotions: Anxious (*F*(1, 833) = 63.10, *p* < .001, *η_p_^2^*= .070), Frustrated: (*F*(1, 833) = 108.87, *p* < .001, *η_p_^2^*= .12), and Confused (*F*(1, 833) = 44.39, *p* < .001, *η_p_^2^*= .051). For norm, we found a statistically significant main effect for frustration (*F*(1, 833) = 9.03, *p* = .003, *η_p_^2^*= .011), while both anxious and confused was not statistically significant (*p* > .05). Importantly, descriptive norms alleviated confusion under induced uncertainty (Norm = yes: M = 4.70, 95% CI [4.45, 4.94], Norm = no: M = 5.06, 95% CI [4.83, 5.31]) and anxiousness under low ambiguity (Norm = yes: M = 2.02, 95% CI [1.84, 2.22], Norm = no: M = 2.33, 95% CI [2.13, 2.56]).


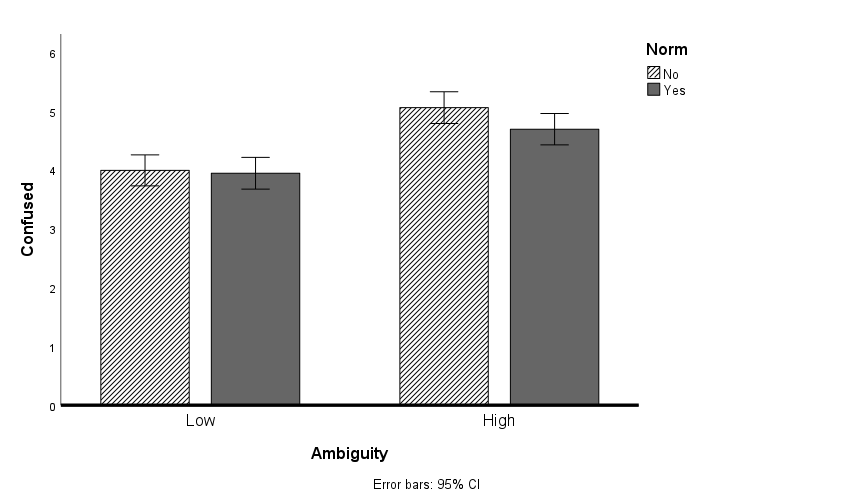


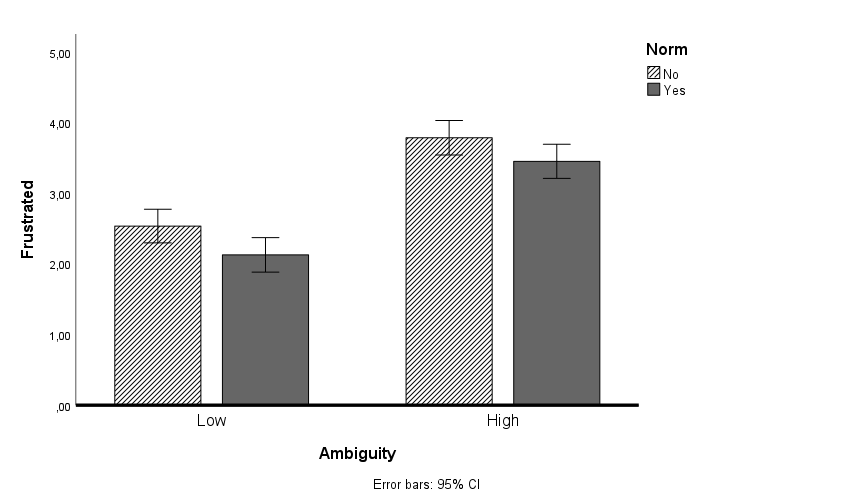


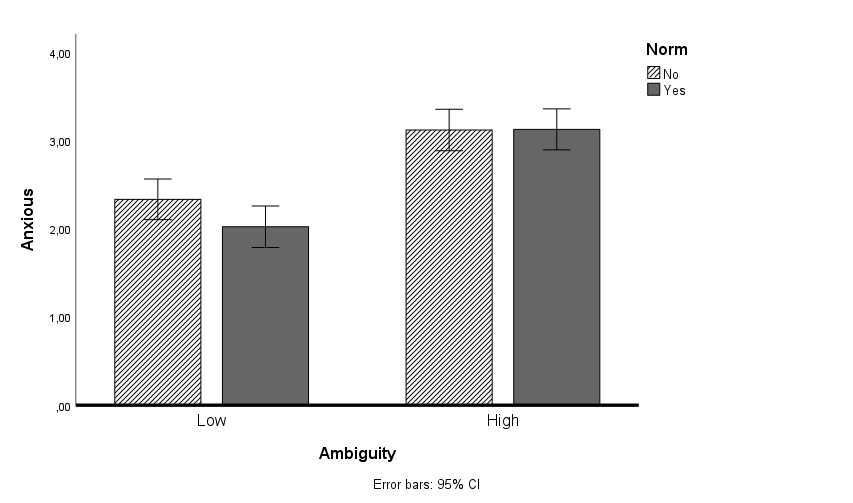


**Appendix D.**

Pilot study 4 for Experiment 3. We tested the choice of hazard prevention support in four scenarios all including risks for hazards (i.e., flood, nuclear accident, heavy storm, and earthquake) in a 2 (uncertainty: risk vs ambiguity) × 2 (norm: yes vs no) mixed-design with repeated measures on the four hazards. 150 participants were recruited from Prolific Academic and paid approximately an hourly salary of 9£ for taking a three-minute survey. The distribution between the choice alternatives in the absence of norms in the risk condition was 23.8% - 76.8% in the flooding scenario, 42.9% - 57.1% in the nuclear accident scenario, 33.3%-66.7% in the heavy storm scenario, and 38.1%-61.9% in the earthquake scenario. Therefore, we chose the nuclear accident scenario for Experiment 3.

**Deviations for the preregistration.**

Hypothesis 2, stating to test the null-hypothesis in the preregistration, was removed from the main analysis as this cannot be tested with NHST. We did however, test this using a Bayesian frequency table. Results showed anecdotal evidence for the alternative hypothesis, BF = 1.24, suggesting no noteworthy main effect of ambiguity, as suggested by the hypothesis. Moreover, the preregistrations suggested using ANOVAS and chi-square tests. Given the limitations of this family of tests, we analyzed or complemented all hypotheses with regression analyses. All models can be found in the R studio codes provided on OSF.
